# Structural Ordering in SWCNT-Polyimide Nanocomposites and Its Influence on Their Mechanical Properties

**DOI:** 10.3390/polym10111245

**Published:** 2018-11-10

**Authors:** Sergey V. Larin, Victor M. Nazarychev, Alexey Yu. Dobrovskiy, Alexey V. Lyulin, Sergey V. Lyulin

**Affiliations:** 1 Institute of Macromolecular Compounds, Russian Academy of Sciences, St. Petersburg 199004, Russia; selarin@macro.ru (S.V.L.); nazarychev@imc.macro.ru (V.M.N.); 2 Faculty of Physics, St. Petersburg State University, Petrodvorets, St. Petersburg 198504, Russia; aleksey_dobrovsky@mail.ru; 3 Theory of Polymers and Soft Matter Group and Center for Computational Energy Research, Department of Applied Physics, Technische Universiteit Eindhoven, PO Box 513, 5600 MB Eindhoven, The Netherlands; a.v.lyulin@tue.nl

**Keywords:** polyimides, structural ordering, crystallization, mechanical properties, nanocomposites, molecular dynamics

## Abstract

Using fully-atomistic models, tens-microseconds-long molecular-dynamic modelling was carried out for the first time to simulate the kinetics of polyimides ordering induced by the presence of single-walled carbon nanotube (SWCNT) nanofillers. Three polyimides (PI) were considered with different dianhydride fragments, namely 3,3′,4,4′-biphenyltetracarboxylic dianhydride (BPDA), 2,3′,3,4′-biphenyltetracarboxylic dianhydride (aBPDA), and 3,3′,4,4′-oxidiphthalic dianhydride (ODPA) and same diamine 1,4-bis[4-(4-aminophenoxy)phenoxy]benzene (diamine P3). Both crystallizable PI BPDA-P3 and two amorphous polyimides ODPA-P3 and aBPDA-P3 reinforced by SWCNTs were studied. The structural properties of the nanocomposites at temperature close to the bulk polymer melting point were studied. The mechanical properties were determined for the nanocomposites cooled down to the glassy state. It was found that the SWCNT nanofiller initiates’ structural ordering not only in the crystallizable BPDA-P3 but also in the amorphous ODPA-P3 samples were in agreement with previously obtained experimental results. Two stages of the structural ordering were detected in the presence of SWCNTs, namely the orientation of the planar moieties followed by the elongation of whole polymer chains. The first type of local ordering was observed on the microsecond time scale and did not lead to the change of the mechanical properties of a polymer binder in considered nanocomposites. At the end of the second stage, both BPDA-P3 and ODPA-P3 PI chains extended completely along the SWCNT surface, which in turn led to enhanced mechanical characteristics in their glassy state.

## 1. Introduction

Semi-crystallizable polymers are widely used in different industrial applications due to their enhanced mechanical properties. The investigation of the polymer crystallization process still remains one of the most complex and intriguing problems in soft matter physics. The structural ordering of such polymers have been intensively studied recently, both experimentally [1,2,3,4] and theoretically [5,6,7,8,9,10,11]. The classical Hoffman–Lauritzen cluster model [12,13] postulates that the crystallization of polymer chains is a single-step kinetic process involving only nucleation. However, the coarse-grained computer simulations of n-alkanes and polyethylene melts show [9,10,11,14,15] that the crystallization process could be more complicated. Yamamoto et al. simulated the crystallization of the polyethylene-like polymers using the united-atoms model [9,10,11]. They showed that polymer chains containing ~130,000 beads each are oriented within rather long few tens of nanoseconds simulation runs. The picture can be even more complex if the external mechanical deformation (as shear flow, for example) has been considered. Anwar et al. [14,15] have shown that the process of crystal growth in the melts of the short n-alkane chains, with and without mechanical shear, is completely different. In general, it was found that there exist two stages that polymers pass through during the crystallization process. First, only the local ordering of small chain fragments is observed. Later, the polymer chains unfold and orient along the same direction during some time. These two stages together could be considered as some initial pre-crystallization process.

The incorporation of the carbon nanofillers in a polymer melt shows a good ability to initialize and even accelerate crystallization, as has already been confirmed for polyethylene [16,17,18], nylon [19,20,21], and for some polyimides (PIs) [22,23,24]. In this case, the difference between the homogeneous and the heterogeneous crystallization is that the carbon nanofiller acts as the crystallization nucleus, where the fragments of the polymer chains begin to order. This ordering is followed by the unfolding of the polymer chains, and, finally, by their layer-by-layer orientation along the nanofiller surface. The experimental methods still could not unambiguously shed light on the polymer structural ordering during the crystallization process. At the same time, the modern computer simulations definitely are cheaper, more convenient, and rather promising to investigate the behavior of polymer chains during the structural ordering and the change of the corresponding polymer mechanical properties.

**Figure 1:**
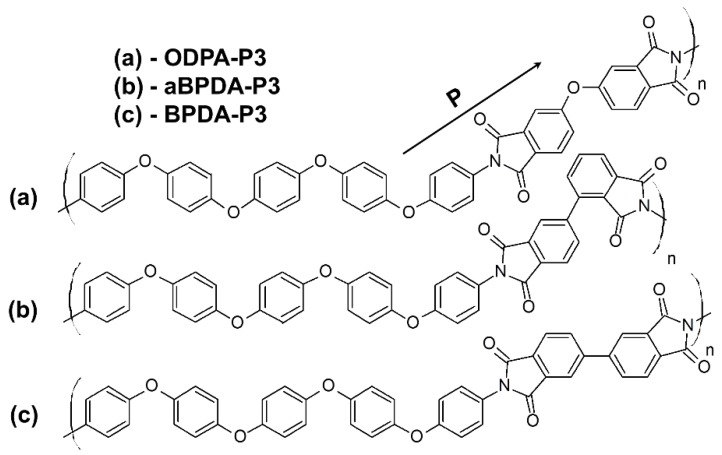
The chemical structure of the repeating units of the polyimides simulated in the present study: ODPA-P3 (**a**), aBPDA-P3 (**b**), and BPDA-P3 (**c**). The arrow marks vector **P** directed along the phthalimide planar moieties of the polyimide chains; its orientation relative to the nanotube axis was investigated. For clarity the vector **P** is shown only for the ODPA-P3 PI; for aBPDA-P3 and BPDA-P3 these vectors have been directed along the same fragments. Reproduced from [34] with permission, copyright Wiley, 2018.

Hegde et al. [25,26] have experimentally investigated three PIs with different dianhydride fragments, namely 3,3′,4,4′-biphenyltetracarboxylic dianhydride (BPDA), 2,3′,3,4′-biphenyltetracarboxylic dianhydride (aBPDA), and 3,3′,4,4′-oxidiphthalic dianhydride (ODPA) and same diamine 1,4-bis[4-(4-aminophenoxy)phenoxy]benzene (diamine P3). Both crystallizable PI BPDA-P3 and two amorphous polyimides aBPDA-P3 and ODPA-P3 have been studied (see Figure 1). The main difference in chemical structure of these polyimides is the additional ether group in the dianhydride fragment of ODPA-P3. The difference between BPDA-P3 and aBPDA-P3 is disparity in the bonding between phthalimide moieties that lead to the formation of a kink in the aBPDA-P3 monomer unit.

The authors [25,26] showed that the initially amorphous ODPA-P3 crystallizes in the presence of the carbon nanotubes, that, in turn, leads to the improvement of its mechanical properties. Hegde et al. [26] found that the structural ordering of the ODPA-P3 PI is even more pronounced as compared to that for the crystallizable BPDA-P3. Despite the fact that the difference between the mechanical characteristics of the unfilled aBPDA-P3, BPDA-P3, and ODPA-P3 samples is rather small, the experimental studies [25,26] have sounded that the embedding of a carbon nanofiller leads to an enhancement of some mechanical properties, such as Young’s moduli and the yield stress for the crystallizable polyimides ODPA-P3 and BPDA-P3 in the glassy state. The improvement of the mechanical properties for both PIs might be caused by the arising of the structural ordering of PI chains induced by the carbon nanotubes. The origin of the effect still remains unclear.

Our recent simulations [27,28,29] have shown that the planar moieties of the crystallizable PI R-BAPB based on the 1,3-bis(3′,4-dicarboxyphenoxy)benzene (dianhydride R) and 4,4′-bis(4′′-aminophenoxy)biphenyl (diamine BAPB) form some ordered structures near both carbon nanotube [28] and graphene [27,29] surfaces. This structural ordering was considered as an initial stage of crystallization (so-called pre-crystallization) of the polymer matrix. In case of graphene-filled nanocomposites, it was shown [27] that the polymer mechanical properties were improved drastically after the appearance of the structural ordering of planar moieties. The anisotropy of the mechanical properties of the R-BAPB polyimide near the graphene surface, depending on the direction of the applied deformation, has been observed as well. Despite rather long simulations reaching the microseconds time scale, the second stage of the chains ordering, i.e., the unfolding and the consequent orientation of the chains as a whole, was not observed. One could expect that much longer simulation times will be required to observe the second pre-crystallization stage, especially in case of long polymer chains with a complex chemical structure. It still remains unclear how the specific stage of the thermoplastic PI crystallization near the surface of the carbon nanofiller (carbon nanotube, for example) can affect their mechanical properties in the glassy state.

However, the two stages of the pre-crystallization process were clearly shown in the simulations using the relatively simple coarse-grained polymer models [9,10,11,14,15]; the insights into the similar process but for polymers having complex chemical structure are of great importance. For example, the additional π–π interactions could influence significantly the crystallization process in case of polyetherimides [30]. The atomistically-detailed simulations of all the pre-crystallization stages could provide an opportunity to determine the specific features of this process in various systems that differ in respect to the nature of the polymer matrix and the nanofiller chemical structure.

The structural properties of aBPDA-P3 and ODPA-P3 polyimides in the presence of a single-walled carbon nanotube (SWCNT) were investigated in our previous study [30]. To describe accurately a difference in the chemical structures of these PIs and to take into account the polymer-nanofiller interactions, the detailed all-atom models were used. The length of the microseconds-long simulations [30] was long enough to qualitatively reproduce the experimental difference between the structural properties of both aBPDA-P3 and ODPA-P3. The simulations have been carried out at *T* = 600 K, close to the melting temperature (*T_m_* ≈ 580 K) of the ODPA-P3-based nanocomposite filled with 0.1 vol % of SWCNT [25,26]. After the three μs-long simulations, we were able to observe a structural ordering of the planar moieties of this polyimide near the SWCNT surface. Such ordering was absent in the simulated aBPDA-P3 samples. Particularly, our findings revealed that the ordering of the ODPA-P3 planar moieties depends mainly on the π–π interactions between the carbon atoms in their heterocyclic fragments and the SWCNT aromatic rings. However, the influence of the SWCNT presence on the mechanical properties of the resulting nanocomposites was not investigated.

The two stages of the pre-crystallization process have been confirmed by the coarse-grained simulations of different *n*-alkanes by Yamamoto et al. [9,10,11] and Anwar et al. [14,15]. One could expect that rather significant changes of the polymer mechanical properties should be observed at the end of the pre-crystallization process, and the full unfolding and the orientation of the PI chains along the SWCNT surface will take place. For such structurally complicated systems as nanocomposites based on the heterocyclic aromatic polyimides, the transition from the local ordering state (the initial stage of the pre-crystallization) to the state where the polymer chains are completely unfolded and are oriented along the nanofiller (the second stage of the pre-crystallization) has not been simulated. The main challenge to atomistically model such a transition is its extremely slow rate, which requires extremely long simulation runs exceeding — by orders of magnitude — the typical nanosecond time scale of the molecular dynamics. In the present study we extend the results of Reference [30] and simulate the structural ordering of the thermoplastic PIs with the main goal to observe the above mentioned possible second stage of the pre-crystallization process and the transition to the state where PI polymer chains are unfolded and oriented.

Based on the previously acquired knowledge, we expect that such kind of the chain orientation and ordering could be observed at temperatures slightly higher than (but close to) the corresponding polymer melting temperature. On the other hand, the simulation timescales required to observe the second pre-crystallization stage are expected to be at least an order of magnitude larger (tens of microseconds) than those (several microseconds) used by us earlier. Such a project demanded more than two-years long continuous modelling using 64 CPU cores on Kurchatov (Intel Xeon E5450) and Lomonosov-2 (Intel Haswell-EP E5-2697v3) supercomputers. As will be shown below, the simulations of such length allow for almost complete unfolding and orientation of PI chains along the SWCNT surface. To the best of our knowledge, such time-consuming all-atom molecular-dynamics simulations have not been performed yet in materials science.

The rest of the paper is organized as follows. The investigated polyimides and the details of the simulation parameters are presented in the Section Models and Simulation Methods. The obtained data are discussed in the Results and Discussions Section. The outputs of the study are summarized in the Conclusions section.

## 2. Models and Simulation Methods

The initial configurations of the ODPA-P3, aBPDA-P3, and BPDA-P3 melts were created using the approach developed previously [29,31,32]. To begin, 27 polymer chains were randomly placed into a periodic cell with rather low overall density. The periodic boundary conditions have been used in all three directions. Each polymer chain consisted of N_p_ = 8 repeating units. Note that the chosen degree of polymerization of the PIs corresponded to the beginning of the polymer regime for the heterocyclic polyetherimides [31]. After that, the SWCNT with a chirality of (5, 5), diameter of 0.7 nm, and length of 4.7 nm was embedded to the center of the simulation cell. The SWCNT model was similar to that used in our previous study and consisted of 420 atoms [28]. Thus, nanocomposites based on aBPDA-P3, BPDA-P3, and ODPA-P3 had 18186, 18186, and 18402 atoms, correspondingly.

The initial nanocomposite samples were compressed to the experimental densities and annealed during three cycles of the stepwise temperature decrease and increase from 800 to 300 K [32]. The total time length of annealing was 72 ns. After that, the melts were cooled down instantly from *Т* = 800 to 600 K, and further simulations were performed during additional 20 μs at *T* = 600 К. The simulated temperature was close enough to the experimental melting point (*T_m_* ≈ 580 K) of the ODPA-P3-based nanocomposite filled with 0.1 vol % of SWCNT [25,26]. At this temperature the ordering of the polymer chains does not break due to the thermal fluctuations. Still, the polymer chains are rather mobile, which allows one to investigate the possible structural transformations [27,28,29,30]. The Berendsen thermostat and barostat [33] were used to keep the temperature and the internal pressure constant with *τ_t_* = 0.1 ps and *τ_p_* = 0.5 ps for the thermostat and for the barostat relaxation times, respectively. All simulations were carried out in *NpT* ensemble with time step 1 fs.

To investigate the influence of the chain orientation on the mechanical properties of the nanocomposites based on aBPDA-P3, ODPA-P3, and BPDA-P3 polyimides, several simulated samples were chosen at different simulation times depending on the polymer chain orientation state. Their initial configurations were chosen from the different parts of the simulated trajectory. Every 150 ns of simulation, seven samples were chosen in the interval from 3 to 4 μs, and another seven configurations were chosen in the interval from 19 to 20 μs. The step-wise cooling of the chosen samples was carried out from the initial temperature *T* = 600 K to the room temperature *T* = 290 K with the cooling rate γ*_с_* = 1.5 × 10^11^ К/min [31,34,35,36,37].

To calculate the mechanical properties, the simulated PI samples were deformed uniaxially at *T* = 290 K using the approach developed earlier [38,39]. All the deformation experiments were carried out with the fixed deformation rate of γ*_d_* = 1.8 × 10^8^ s^−1^ [27,38,40]. The transversal box directions were kept at atmospheric pressure during the deformation procedure.

The diagonal components of the stress tensor *P_i_*, *i =* {*x, y, z*} and the dimensions *L_i_* of the periodic cell were calculated every picosecond. These values were used to produce the stress-strain dependence as [39]

(1) $$\begin{array}{l} \sigma =-P_{i},\\ \varepsilon =\frac{L_{i}-L_{0i}}{L_{0i}}, \end{array}$$

where $L_{0i}$ is the cell dimension at the beginning of the deformation (t = 0). Initially, the dependence *σ(ε)* was very close to linear. This linear elastic regime was clearly observed up to ~2% of the deformation *ε* [39] and was used to extract the Young’s modulus *E* as

(2) σ=Eε. 

The yield stress value *σ_y_* was determined from σ*(*ε*)* as the average stress value where the plastic deformation occurs without a significant increase in a load (i.e., without significant change of stress value).

The Gromacs MD software package (version 5.0.5) [41,42] was used to perform all the simulations with the help of the Gromos53a5 force field [43,44]. In our previous studies it was shown that this force field allows to investigate the structural and thermomechanical properties of different thermoplastic PIs. The simulated results were in a good agreement with the experimental data [28,31,32,34,38]. It was also shown previously that the partial charges have no significant influence on the structural and mechanical properties of the considered thermoplastic PIs [30,34]. To speed up the simulations, the electrostatic interactions have not been taken into account in the current study; the corresponding partial charges were put to zero.

## 3. Results and Discussions

In the previous studies [27,28,29,30], we analyzed the orientation of the phthalimide planar moieties in the PI chains. This orientation was represented by the **P** vector (see Figure 1) relative to the nanofiller surface and was used to characterize the local ordering of the polyimide chains in the vicinity of the carbon nanofiller surface. The orientation angle θ between the vector **P** directed along the phthalimide moiety and the carbon nanotube axis was determined, and the corresponding order parameter *S*(*r*) was calculated

(3) S(r)=32〈cos2θ(r)〉−12, 

where *r* is the distance from the nanotube axis to the polymer chain planar moiety.

**Figure 2:**
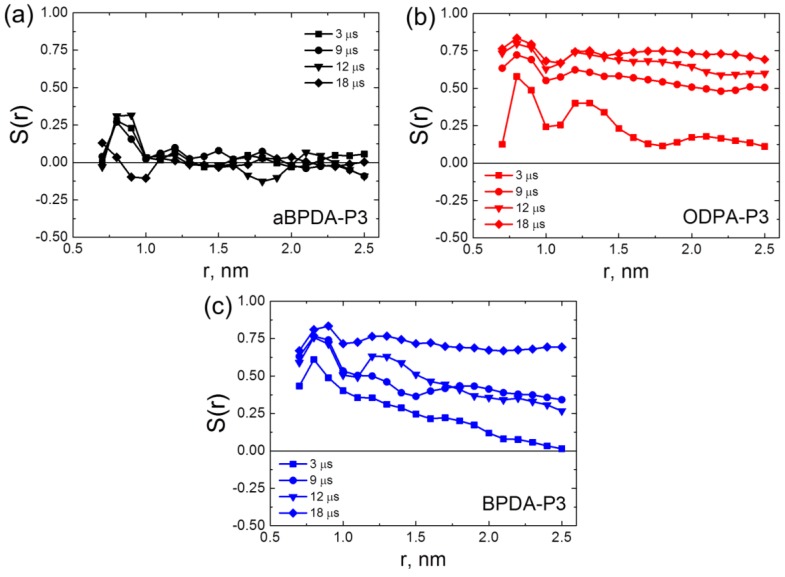
The order parameter *S*(*r*) for the orientation angle *θ* of the polymer-chain planar moieties relative to the single-walled carbon nanotube (SWCNT) axis in nanocomposites based on aBPDA-P3 (**a**), ODPA-P3 (**b**), and BPDA-P3 (**c**) at Т = 600 К as functions of the distance from the nanotube axis after different simulation times shown in the legends. The results for the ODPA-P3 and aBPDA-P3 polyimides (PIs) calculated after 3 μs of simulation are taken for comparison from our previous study [30].

To determine how the local orientation ordering possibly changes during very long simulation run, in the present simulations we calculated the order parameters at different simulation times, Figure 2.

After 3-μs simulation run the order parameters *S*(*r*) showed that planar moieties of BPDA-P3 and ODPA-P3 were oriented along the axis of SWCNTs in the vicinity of nanotube surface, and the slow decrease of the *S*(*r*) dependence was observed with the increase of the distance from the nanotube axis. In all nanocomposites the first peak in the *S*(*r*) dependence corresponds to the relatively high ordering of the planar moieties along the SWCNT surface in the vicinity of the nanofiller surface (so called subsurface layer). Contrast to that, in the nanocomposite samples based on the amorphous aBPDA-P3, the order parameter is close to zero everywhere except the subsurface layer. This means that in aBPDA-P3-based nanocomposite no long-range order exists, and the polymer remains amorphous. These results are in full agreement with the experimental data of Hedge et al. [25,26]. After three microseconds of the simulation the second peak that corresponds to the ordering of planar moieties further from the SWCNT surface was observed only for ODPA-P3. This might indicate that ODPA-P3 monomers have a larger degree of orientation near the SWCNT surface, as compared even to that for the crystallizable BPDA-P3. The results obtained confirm that after 3-μs simulations, only the initial crystallization stage is observed and only rather small fragments of polymer chains form some oriented structures.

In the course of the production run, beyond 3 μs of simulation, the gradual increase of the order parameter takes place for both BPDA-P3 and ODPA-P3. The first peak that corresponds to the subsurface polymer layer increases slightly with time, but the more pronounced growth of the order parameter was found for the distances r > 1 nm far from the SWCNT surface. After 19 μs of simulations the order parameters *S*(*r*) in nanocomposites based on BPDA-P3 and ODPA-P3 preserve almost constant values close to 0.8 upon increasing the distance from the SWCNT surface, Figure 2. This allows us to conclude that the distribution of the orientation and the ordering of the planar moieties of PI chains across the simulation cell occurs on this time scale. The obtained results are in good qualitative agreement with experimental data of Qiang et al. [45,46], where the increase of Herman’s orientation factor upon increase of time has been found for different block copolymers during aligning.

**Figure 3:**
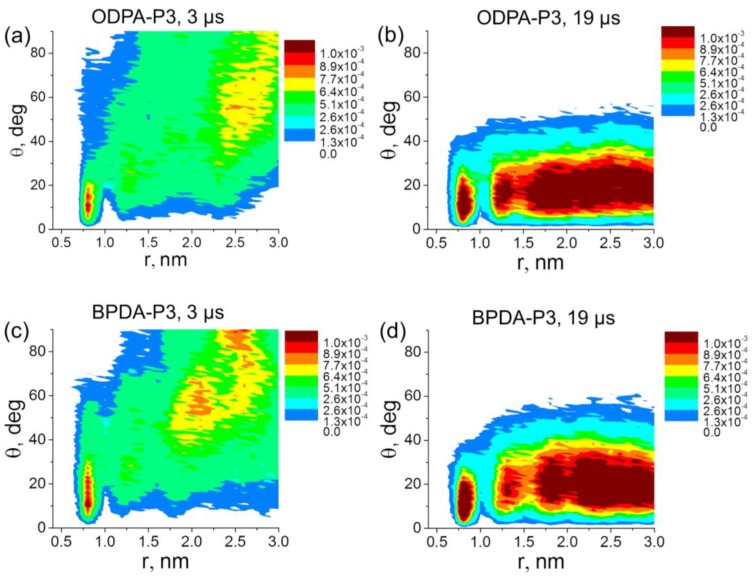
The distribution of the orientation angles *θ* for the vector **P** relative to the SWCNT axis in nanocomposites based on ODPA-P3 (**a**,**b**) and BPDA-P3 (**c**,**d**) after 3 μs (**a**,**c**) and 19 μs (**b**,**d**) of simulation. The results for ODPA-P3 have been calculated after 3 μs of simulation and are taken for comparison from our previous study [30].

The obtained results are supported by the analysis of the distribution of the orientation angle θ between the vector **P** and the SWCNT axis. After 3 μs of simulation, the orientation angle distributions (see Figure 3a,c) demonstrate that the planar moieties of PI chain in ODPA-P3 and BPDA-P3-based nanocomposites were stacked parallel to each other only near the surface of the SWCNT nanofiller. Note that the distributions in Figure 3 show the fraction of planar moieties of PI chains that have given θ value at given *r* value. So, the values in the legends in Figure 3 are dimensionless.

After 19 μs of simulation, both BPDA-P3 and ODPA-P3 exhibited the maxima in the distribution diagrams of the angles θ in the range of 5–15 degrees. Meanwhile, the structural ordering of the planar moieties extended to a distance of about 3 nm from the SWCNT axis (Figure 3b,d), that is approximately half of the periodic cell size. The similar character of distributions of the orientational angles for the nanocomposites based on ODPA-P3 and BPDA-P3 is due to the structural similarity of the planar moieties of both PI chains that were chosen to determine θ.

**Figure 4:**
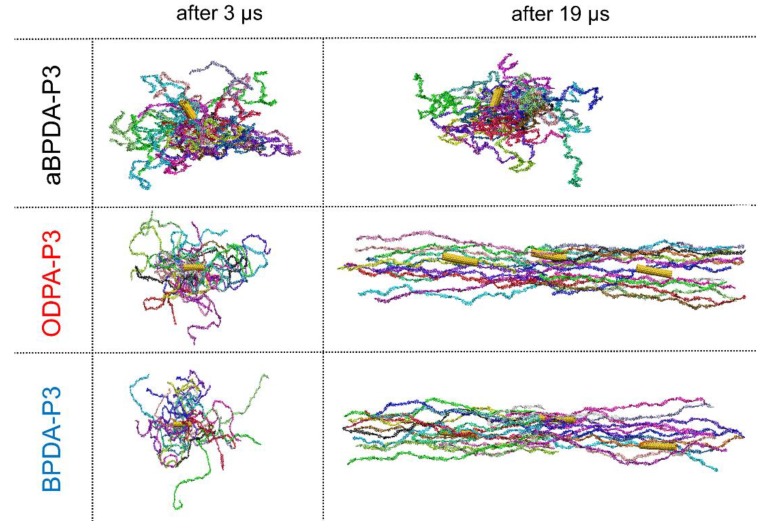
The instant configurations of a single periodic cell of the SWCNT-PI nanocomposites based on aBPDA-P3 (**top**), ODPA-P3 (**middle**), and BPDA-P3 (**bottom**) after 3 and 19 microseconds of simulation. The periodic boundary conditions were removed and polymer chains are drawn continuously to show the conformation of PI chains in each system. For the systems where polymer chains are unfolded and oriented, two additional periodic images of SWCNT are shown which are in contact with the PI chains depicted. Each polymer chain is shown in its own color. The SWCNT is shown in yellow on all the snapshots.

The analysis of the instant configurations of the composites after 3 and 19 μs of simulation (Figure 4) suggests that BPDA-P3 and ODPA-P3 chains change their conformation from the coiled one to some extended shape upon development of the ordering in these nanocomposites, i.e., PI chains start their unfolding which correspond to the chain orientation along the SWCNT surface. At the same time, the amorphous aBPDA-P3 polymer chains do not change neither conformation, nor orientation near the SWCNT surface, Figure 4. Thus, aBPDA-P3 stays amorphous in the corresponding nanocomposites in full agreement with the experimental [25,47] and the simulation [30] results.

**Figure 5:**
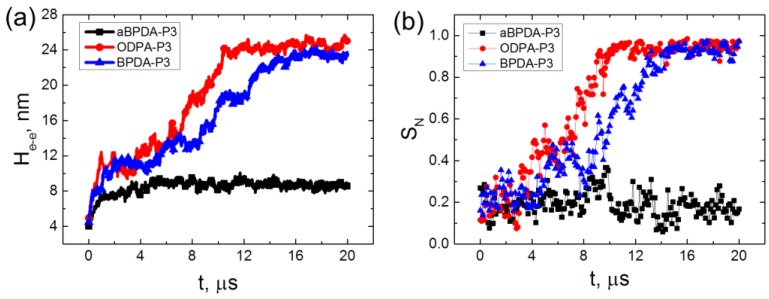
The time dependence of (**a**) the end-to-end distance (H_e-e_) and (**b**) the nematic order parameter *S_N_* for aBPDA-P3, ODPA-P3, and BPDA-P3 in nanocomposites with SWCNT.

The average end-to-end distance (H_e-e_) and the nematic chain order parameter *S_N_* of three considered PIs have been calculated (Figure 5) to confirm the unfolding and orientation of the PI chains during the 20 μs-long simulation. *S_N_* is determined as the largest eigenvalue of the order tensor $S_{\alpha \beta }$

(4) $$S_{\alpha \beta }=\frac{1}{N_{ch}}\sum_{i=1}^{N_{ch}}\frac{3}{2}{\bar{u}}_{i\alpha }{\bar{u}}_{i\beta }-\frac{1}{2}\delta_{\alpha \beta },\;$$

where $N_{ch}$ is the number of chains for which the calculation is performed, ${\bar{u}}_{i}$ is the unit vector parallel to the end-to-end vector of i-th chain, δ is the Kronecker delta, and *α, β = x*, *y,* or *z*.

The end-to-end distance H_e-e_ for all three considered polyimides in composites behave almost identically up to simulation times close to ~4 μs. It grows from relatively small values corresponding to the collapsed state after the initial compression and annealing procedures to the values close to the theoretical predictions for H_e-e_ of a polymer coil in melt [32]. However, it should be noted that the end-to-end distances H_e-e_ for ODPA-P3 and BPDA-P3 polyimides are slightly higher than those for aBPDA-P3. This is due to the local ordering of ODPA-P3 and BPDA-P3 chains in nanocomposites that leads to the change of the average size of PI chains in the systems considered.

In case of the amorphous aBPDA-P3, no further increase of the polymer chain size was observed after 3 μs of simulation. However, for ODPA-P3 and BPDA-P3 the value of H_e-e_ starts to increase further after ~4 μs of simulation till H_e-e_ reaches the value close to the theoretical end-to-end distance for a fully unfolded chain (26 and 27 nm for BPDA-P3 and ODPA-P3, correspondingly).

At the same time, the significant increase of the nematic order was observed in the nanocomposites based on the ODPA-P3 and BPDA-P3. This allows us to conclude that in these systems, the corresponding polymer chains unfold and orient simultaneously in the direction of the SWCNT axis. Such behavior can be considered as the polymer “pre-crystallization” stage following the orientation of the planar moieties near the CNT surface, as was shown in our previous study [30]. The unfolding and the orientation of the polymer chains finish after about 15 μs of simulation, and the order parameter *S_N_* values become larger than 0.9. After that time, the end-to-end distance and the nematic order parameter do not change much during rather long simulation time (at least of the order of 5 μs).

It should be noted that some fluctuations observed on the curves at Figure 5 are mostly due to thermal motion of polymer chains in the systems studied. Fluctuations in the region where transition from the coiled conformations of chains to extended ones happen (from 4 to 15 µs in case of nanocomposites based on ODPA-P3 and BPDA-P3 polyimides) could be related to nonstationary character of this process.

The results obtained confirm the proposed two-stage model of polymer pre-crystallization. During the first stage only the local ordering of some planar moieties of PI chains occur in the vicinity of the nanofiller surface. At this stage the polymer chains preserve a coiled conformation and are not oriented as a whole. This state lasts for several microseconds before unfolding and orientation of the chains started. The second stage is the transition of the PI chains to the unfolded and oriented state that occurs during approximately 10 μs for the chains of the simulated polymerization degree *N_p_* = 8 at the simulated temperature of *T* = 600 K. At this second stage, the order degree in the system grows, and the ordering of the polymer chains spreads across the whole simulation cell.

We turn to the second question of the present study, and the influence of the structural ordering stage in the considered nanocomposites on their mechanical properties in a glassy state were studied next. To determine the mechanical properties of nanocomposites with different ordering, we considered samples taken after 3 μs of simulation time where only the orientation of the PI chain fragments were observed and after 19 μs of simulation where all the PI chains are unfolded and are oriented along the SWCNT.

Again, seven different configurations of three PIs have been chosen from the simulation trajectory in two intervals from 3 to 4 μs and from 19 to 20 μs to determine the corresponding mechanical properties. These time intervals correspond to the two stages of the pre-crystallization process observed in the ODPA-P3 and BPDA-P3-based nanocomposites.

These samples have been cooled down from T = 600 K to T = 290 K with a cooling rate γ_c_ = 1.5 × 10^11^ K/min. The simulated density in a glassy state for a crystallizable BPDA-P3 and for an amorphous ODPA-P3 was slightly higher than that for the samples of the same polyimides but taken before unfolding and orientation of the PI chains started, i.e., the transition to unfolded and oriented state leads to the additional compaction of a polymer matrix in the nanocomposites. At the same time, no difference between the densities of amorphous BPDA-P3 samples that were cooled down after 3 μs or after 19 μs was observed.

**Figure 6:**
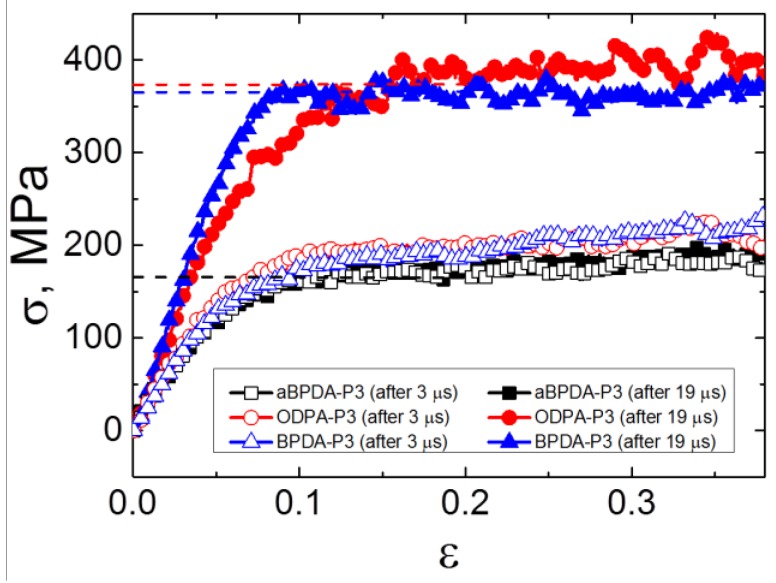
The stress-strain dependence for the simulated nanocomposites based on ODPA-P3, aBPDA-P3, and BPDA-P3 polyimides after 3 and 19 μs of simulation. The dashed lines indicate the yield stress values of considered nanocomposites after 19 μs of simulation.

The cooled down samples were deformed at T = 290 K. The stress-strain curves of the samples after 3 μs (before PI chains unfolding) and after 19 μs (after unfolding and orientation of the chains as a whole) of simulation were obtained, Figure 6. The Young’s moduli and yield stress values have been calculated and summarized in Table 1. The yield stress values were calculated as average stress in the region of load (from 0.1 to 0.2 of strain). The mechanical properties of the unfilled polyimides obtained in our previous study [34] are shown there for comparison. It should be noted that the deformation procedure was performed for the systems that comprise polymer matrix and SWCNT. As in the present simulations, the relatively short nanotubes were used, and they did not impede the simulation of deformation, in contrast to the graphene nanoflake of infinite size that was used in our previous study [29]. In the latter case, some additional steps had to be performed to determine the mechanical properties of the polymer matrix in a nanocomposite, including graphene removal and the additional compression of a polymer matrix.

The analysis of the obtained mechanical properties suggests that the Young’s moduli and the yield stress values increase significantly only after the transition to the state which is characterized by the entirely unfolded and oriented PI chains in nanocomposites based on ODPA-P3 and BPDA-P3. At the same time, there is no difference in the simulated stress-strain behavior for all three PIs after 3 μs of simulation, as well as no difference in the mechanical properties of the aBPDA-P3 after 3 μs and 19 μs of simulation time. Moreover, the comparison of the mechanical properties of nanocomposites with those for the unfilled samples [34] show no difference between the properties of pure polymers and the corresponding nanocomposites in the first pre-crystallization stage before the transition to the unfilled and oriented state of chains, as could be seen from Table 1.

Thus, we can conclude that the local structural ordering of the planar moieties of PI chains near the SWCNT surface (i.e., the first stage of the pre-crystallization process) does not influence significantly the mechanical properties of the simulated nanocomposites. The Young’s moduli and the yield stress values for the samples after 3 μs of simulation (only local segmental ordering is found at this stage) were similar for all the nanocomposites considered and were close to the values obtained for the amorphous unfilled samples of all three PIs. The second stage of crystallization was characterized by the unfolding and orientation of PI chains and occurred in nanocomposites based on ODPA-P3 and BPDA-P3 polyimides, making a big difference, and the corresponding mechanical characteristics increased drastically.

## 4. Conclusions

In the present study, 20-microsecond molecular-dynamic simulations were performed to investigate the structural and mechanical properties of nanocomposites based on the polyetherimides BPDA-P3, ODPA-P3, and BPDA-P3 filled with single-walled carbon nanotubes.

For the first time, the two distinguished stages of a polymer matrix structural ordering were found using the fully-atomistic models for the description of such complex systems at temperatures slightly above the corresponding experimental melting point. At the first stage observed on the microsecond time scale, local segmental ordering was confirmed. At this stage, only relatively short PI chains fragments were oriented along the SWCNT axis in the vicinity of the nanofiller surface. During this stage, the polymer chains had coiled conformations and were not oriented as a whole in the direction of the SWCNT axis. This kind of ordering could be considered as the initial stage of the polymer matrix pre-crystallization in the simulated nanocomposites.

The second stage of the pre-crystallization starts when the individual polymer chains unfold and orient in the same direction. These orientation and ordering effects are accompanied by the increase of the chains end-to-end distance and the increase of the chain nematic order parameter. During this stage, all chains in the periodic cell form highly ordered structures and align in the direction of the SWCNT axis. The time of the unfolding and of the orientation of polymer chains as a whole is of the order of 10 μs for the considered PI polymerization degree and simulated temperature. This time is several times longer than what is necessary to observe the local ordering of the planar PI moieties near the SWCNT surface.

The analysis of the simulated data has shown that the local orientation of the planar moieties near the SWCNT surface does not result in the change of the mechanical properties, as compared to those for the corresponding unfilled PI samples. It is the unfolding and the orientation of PI chains as a whole in the BPDA-P3 and ODPA-P3-based nanocomposites that leads to the large increase of the Young’s moduli and the yield stress values for the materials studied. Thus, we could suggest that the embedding of carbon nanotubes may initiate the structural ordering of heterocyclic polymers on the scale of the whole chains. This large-scale structural ordering plays the main role in the improvement of the mechanical properties of nanocomposites reinforced by the carbon nanotubes.

It should be noted that the highest ordering degree possible in nanocomposites simulated at the temperatures higher than polymer matrix melting point was obtained in the present study. It is not likely that the further simulation will lead to additional change of polymer chains ordering and overall mechanical properties of nanocomposites. However, the ordering observed does not correspond to the crystalline structure of polyimides considered and the crystallization of a polymer could lead to the higher mechanical properties than those obtained in the presented study. In order to observe the crystallization of a polymer and its influence on the nanocomposite mechanical properties it would be necessary to decrease the simulation temperature well below the melting point, and perform additional simulation on the tens of microseconds timescale. Such a work could be the subject of the future study.

## Figures and Tables

**Table 1:** The Young’s moduli and yield stress values for the simulated PIs aBPDA-P3, ODPA-P3, and BPDA-P3 after 3 and 19 μs of simulations (before and after the structural transition to the unfolded states). The results for the unfilled samples (without SWCNT) were calculated using the samples obtained in our previous study [34].

PI	Young’s Modulus, GPa			Yield Stress, MPa		
	w/o SWCNT (After 3 μs)	with SWCNT (After 3 μs)	with SWCNT (After 19 μs)	w/o SWCNT (After 3 μs)	with SWCNT (After 3 μs)	with SWCNT (After 19 μs)
aBPDA-P3	3.0 ± 0.1	3.1 ± 0.2	2.7 ± 0.6	178 ± 6	168 ± 6	169 ± 6
ODPA-P3	3.1 ± 0.2	3.2 ± 0.1	4.5 ± 0.6	182 ± 5	189 ± 5	368 ± 18
BPDA-P3	3.1 ± 0.2	3.1 ± 0.2	5.0 ± 0.6	177 ± 8	187 ± 6	362 ± 10
